# Serological evidence of human infection with *Pteropine orthoreovirus* in Central Vietnam

**DOI:** 10.1002/jmv.24274

**Published:** 2015-06-02

**Authors:** Harpal Singh, Masayuki Shimojima, Thanh Cao Ngoc, Nguyen Vu Quoc Huy, Tran Xuan Chuong, An Le Van, Masayuki Saijo, Ming Yang, Masami Sugamata

**Affiliations:** ^1^ Department of Intelligent Mechanical Systems, Graduate School of System DesignTokyo Metropolitan UniversityTokyoJapan; ^2^ Department of Virology 1National Institute of Infectious DiseasesTokyoJapan; ^3^ Department of Obstetrics and GynecologyHue University of Medicine and Pharmacy Hue CityVietnam; ^4^ Department of Infectious DiseasesHue University of Medicine and PharmacyHue CityVietnam; ^5^ Department of MicrobiologyHue University of Medicine and PharmacyHue CityVietnam; ^6^ Department of Hygiene and Public Health, Graduate School of Human Health Sciences Tokyo Metropolitan UniversityTokyoJapan

**Keywords:** seroepidemiology, Hue City, Central Vietnam, ELISA, neutralization test, indirect immunofluorescence assay

## Abstract

*Pteropine orthoreovirus*, potentially of bat origin, has been reported to cause respiratory tract infections among human beings in Southeast Asia. Twelve IgG ELISA‐positive cases with antibodies against *Pteropine orthoreovirus* were detected among 272 human serum samples collected between March and June 2014 from in and around Hue City, Central Vietnam. These 12 cases were IgM ELISA negative. Neutralizing antibodies were also detected among six of these cases with the highest titer of 1:1,280 in 2 cases (both female, 32 and 68 years old, respectively). This is the first report of human infection with *Pteropine orthoreovirus* in Central Vietnam. These findings indicate the need for surveillance on *Pteropine orthoreovirus* infections in Southeast Asia to enable prevention and control strategies to be developed should a change in virulence occur. ***J. Med. Virol. 87:2145–2148, 2015*.** © 2015 The Authors. *Journal of Medical Virology* Wiley Periodicals, Inc.

## INTRODUCTION

Infectious disease surveillance systems in Central Vietnam are limited because of the absence of antibody detection testing against causative viral diseases. The determination of the prevalence of viral diseases and its epidemiology are crucial in the development of prevention and control strategies for infectious diseases in Central Vietnam.

Over the past few years, the isolation of novel respiratory tract infectious agents, specifically those of family *Reoviridae*, subfamily *Spinareovirinae*, genus *Orthoreovirus*, species *Pteropine orthoreovirus* (PRV) [Chua et al., 2007, 2008, 2011; Cheng et al., 2009; Voon et al., 2011; Wong et al., 2012; Yamanaka et al., 2014], which show close genetic relationship to bat‐borne orthoreoviruses [Gard and Marshall, 1973; Pritchard et al., 2006; Du et al., 2010; Thalmann et al., 2010; Voon et al., 2011; Hu et al., 2014; Lorusso et al., 2015]; among human beings living in or travelling to Southeast Asia specifically Malaysia and Indonesia suggests the potential of continuous spill‐over events into human populations [Chua et al., 2007, 2008, 2011; Cheng et al., 2009; Wong et al., 2012; Yamanaka et al., 2014]. Although, there is no direct evidence of bat origin in these human cases, the risk factor of exposure to bats before the onset of disease suggests otherwise [Chua et al., 2007, 2008; Wong et al., 2012; Yamanaka et al., 2014]. Moreover, the contribution of these viral reservoirs to the potential evolution of these viruses indicates the importance for these infections to be monitored.

This study aimed to determine the existence of PRV infections in Hue City, Central Vietnam owing to the importance of the biological nature and unknown virulence potential of PRV infections in Southeast Asia.

## MATERIALS AND METHODS

### Sample Collection

A total of 272 serum samples from patients living in and around Hue City who visited the Outpatient Department of the Hue University Hospital complaining of nonspecific symptoms between March and June 2014 were collected, processed, and used in the subsequent serological tests.

### Ethical Statement

Serum samples collected from all patients were carried out under informed consent. All protocols and procedures were approved by the Research and Ethical Committees for the use of human subjects of the Hue University of Medicine and Pharmacy.

### Antibody Detection Tests

Three different serological tests for the detection of antibodies to PRV were utilized in this study consisting of (test[antigen used]): IgM and IgG ELISA for the detection of antibodies to PRV (recombinant baculovirus expressed major outer capsid protein of Miyazaki‐Bali/2007 PRV), neutralization test for the detection of the presence of antibodies that neutralize the infectivity of PRV (Miyazaki‐Bali/2007 PRV) and immunofluorescence assay (IFA) to detect the presence of antibodies to PRV (Miyazaki‐Bali/2007 PRV) described in the following sections. For negative controls, serum collected from healthy volunteers was used.

### ELISA

The antigen used for IgM and IgG ELISA consisted of the recombinant major outer capsid protein, a conservative protein among the different reported PRV strains, of the Miyazaki‐Bali/2007 PRV strain which was expressed using a baculovirus expression system as previously described [Fukushi et al., 2012]. The recombinant baculovirus without the polyhedrin gene (Δp) served as the negative antigen for each test sample. The standard ELISA protocol was followed as previously described [Fukushi et al., 2012] using serum diluted fourfold from 1:100 to 1:6,400. The means and standard deviations were determined from serum specimens collected from healthy volunteers. The cutoff value for the assay was defined as the mean plus three standard deviations at each dilution point.

### Neutralization Test

The 12 ELISA‐positive serum samples were heat‐inactivated (56°C for 30 min) and diluted fourfold with Dulbecco's Modified Eagles Medium (DMEM) containing 2% Fetal Calf Serum (FCS) from 1:20 to 1:5,120. Each test sample (50 μl by volume) was then mixed with the same volume of DMEM containing Miyazaki‐Bali/2007 PRV strain at an infectious dose of 200 plaque forming units per 50 μl and the mixture was incubated for 1 hr at 37°C for neutralization. After incubation, the mixtures were tested for neutralization by cytopathic effect (CPE) inhibition assay using Vero cells. The neutralization titer value was defined as the concentration of serum to reduce the number of plaques by 50% compared to the serum‐free virus controls.

### Immunofluorescence Assay (IFA)

IFA antigen slides were prepared using Miyazaki‐Bali/2007 PRV infected to uninfected Human Embryonic Kidney cells (HEK 293T) [ATCC no. CRL‐3216] at ratio of 1:3. Serum samples were diluted twofold with PBS from 1:20 to 1:640 and 20 μl of the said mixture was spotted over each IFA slide well followed by incubation for 1 hr at 37°C. The slides were then washed with PBS, and then the wells were spotted with goat anti‐human IgG antibody—FITC conjugated (1:200 dilution; Zymed Laboratory). IFA was not done for samples with neutralization titers of <1:20.

## RESULTS

Twelve positive cases with IgG antibodies against PRV were detected among the 272 serum samples with IgG titers of 1:100 (1 case), 1:400 (5 cases), 1:1,600 (4 cases), and 1:6,400 (2 cases) (Table I). These consisted of 7/12 females while 5/12 were males. Among the ELISA positives, six showed the presence of neutralizing antibodies with neutralizing titers of 1:20 (2 cases), 1:80 (1 case), 1:320 (1 case), and 1:1,280 (2 cases). The highest titer of neutralizing antibodies was observed in the 32 and 68‐year‐old female patients', respectively. Nonspecific symptoms at time of presentation of these patients are described in Table I.

**Table I:** *Pteropine orthoreovirus* Antibody‐Positive Cases from Patients in and around Hue City, Central Vietnam Collected between March and June 2014

			Antibodies			
			ELISA			
No.	Age	Sex^a^	IgM	IgG	Neutralization test	Immunofluorescence assay
1.	16	F	<1:100	1:400	<1:20	ND^b^
2.	20	F	<1:100	1:6,400	<1:20	ND
3.	20	M	<1:100	1:1,600	1:80	1:20
4.	24	M	<1:100	1:400	<1:20	ND
5.	27	F	<1:100	1:1,600	<1:20	ND
6.	32	F	<1:100	1:1,600	1:1,280	1:160
7.	38	M	<1:100	1:6,400	<1:20	ND
8.	38	F	<1:100	1:400	<1:20	ND
9.	50	M	<1:100	1:100	1:20	1:10
10.	56	F	<1:100	1:400	1:20	1:10
11.	57	M	<1:100	1:400	1:320	1:80
12.	68	F	<1:100	1:1,600	1:1,280	1:160

Chief complaints: fever with headache (No. 3, 5, 8, 9, and 11); fever with skin rash (No. 4); fever with headache and loss of appetite (No. 7); fever with malaise (No. 10); abdominal pain (No. 1); epigastric pain (No. 2); cough (No. 12), and not known (No. 6).

^a^ M: Male, F: Female ^b^ND: Not done.

## DISCUSSION

Reovirus, a double strand (ds) RNA virus, was first isolated from humans in the 1950s [Dermody et al., 2013]. The virus usually causes asymptomatic infections if not minimal respiratory and gastrointestinal tract symptoms among humans. There has been increasing reports recently of PRV causing acute respiratory tract infections [Chua et al., 2007, 2008, 2011; Cheng et al., 2009; Voon et al., 2011; Wong et al., 2012; Yamanaka et al., 2014] in humans with epidemiological links to Southeast Asia such as Miyazaki‐Bali/2007 PRV [Yamanaka et al., 2014], Melaka virus [Chua et al., 2007], Kampar virus [Chua et al., 2008], HK46886/09 [Wong et al., 2012], HK50842/10 [Wong et al., 2012], HK23629/07 [Cheng et al., 2009; Wong et al., 2012], and Sikamat/MYS/2010 [Chua et al., 2011]. These human pathogenic strains show a strong phylogenetic relationship to orthoreoviruses of bat origin isolated from Europe, Southeast Asia, and Australia such as Broome virus [Thalmann et al., 2010], Nelson Bay virus [Gard and Marshall, 1973], Pulau virus [Pritchard et al., 2006], Xi River virus [Du et al., 2010], Cangyuan virus [Hu et al., 2014], and Indonesia/2010 [Lorusso et al., 2015]. Although nonspecific symptoms have been reported from patients infected with different PRV strains with epidemiological links to Malaysia and Indonesia—both locally acquired [Chua et al., 2007, 2008, 2011] and one imported case from Indonesia to Japan [Yamanaka et al., 2014] and three imported cases from Indonesia to Hong Kong [Cheng et al., 2009; Wong et al., 2012]—the probability of reinfection with this virus in these cases and in this present study cannot be excluded. Thus, further investigation of the relationship of the symptoms experienced by the patients in this study and the presence or absence of neutralizing antibodies with PRV infection should be carried out in the future.

The genetic diversity and evidence of the exposure to—or the presence of—bats in the environment(s) of the patient(s) infected with the Melaka [Chua et al., 2007], Kampar [Chua et al., 2008], and Sikamat/MYS/2010 [Chua et al., 2011] strains is suggestive of the continued evolution and possibly virulence of PRV and its potential spillover from bats to humans [Chua et al., 2007, 2008, 2011; Cheng et al., 2009; Wong et al., 2012; Yamanaka et al., 2014] prompting the need for more serological and virological surveillance of this virus among humans and bats in Southeast Asia to better understand its geographic distribution and to monitor its virulence. The emergence of PRV causing nonfatal respiratory illness in humans with as of yet limited human‐to‐human transmission [Chua et al., 2008, 2011; Yamanaka et al., 2014] presents a model of the possibility of occurrence of other novel viruses, with greater virulence potential. These warrants further research into the mode of infection, disease transmission, social, environmental, and economic risk factors in guiding the development of surveillance, risk assessment, and public health and medical response to PRV and similarly, other viruses.

Knowledge gained from the continued occurrence of viral diseases has shown that the proper understanding of viral dynamism and evolution of viral species and the contribution of viral reservoirs and hosts in the natural evolution of the viruses and virulence require consistent monitoring. This will enable response strategies to be developed and implemented rapidly should a change in viral virulence occur.

The first report of human infection with PRV in Central Vietnam is presented in this study highlighting the need for prospective surveillance of PRV infections in Southeast Asia to monitor its nature.
